# Gut Microbiota as Important Mediator Between Diet and DNA Methylation and Histone Modifications in the Host

**DOI:** 10.3390/nu12030597

**Published:** 2020-02-25

**Authors:** Patrizia D’Aquila, Laurie Lynn Carelli, Francesco De Rango, Giuseppe Passarino, Dina Bellizzi

**Affiliations:** 1 Department of Biology, Ecology and Earth Sciences (DIBEST), University of Calabria, 87036 Rende, Italy; f.derango@unical.it (F.D.R.); giuseppe.passarino@unical.it (G.P.); dina.bellizzi@unical.it (D.B.); 2 Medical Laboratory, 87100 Cosenza, Italy; laurielynncarelli@gmail.com

**Keywords:** gut microbiota, microbiome, diet, DNA methylation, histone modification, host epigenome

## Abstract

The human gut microbiota is a complex ecosystem consisting of trillions of microorganisms that inhabit symbiotically on and in the human intestine. They carry out, through the production of a series of metabolites, many important metabolic functions that complement the activity of mammalian enzymes and play an essential role in host digestion. Interindividual variability of microbiota structure, and consequently of the expression of its genes (microbiome), was largely ascribed to the nutritional regime. Diet influences microbiota composition and function with short- and long-term effects. In spite of the vast literature, molecular mechanisms underlying these effects still remain elusive. In this review, we summarized the current evidence on the role exerted by gut microbiota and, more specifically, by its metabolites in the establishment of the host epigenome. The interest in this topic stems from the fact that, by modulating DNA methylation and histone modifications, the gut microbiota does affect the cell activities of the hosting organism.

## 1. Introduction

There are 10–100 trillions of microbes that live symbiotically with humans constituting an ecosystem of bacteria, viruses, archaea, and fungi [1,2,3]. Collectively, these microscopic organisms form the human microbiota that is located on the skin, in the mouth, gut, and on other mucosal surfaces. Similarly, the term microbiome refers to the genes these cells harbor, whose number is about 100 times greater than that of the human genome [4,5,6,7]. The component of the microbiota which has attracted the most interest of researchers is hosted in the gastrointestinal tract, to date intensely studied for its deep connection with various aspects of development and life of the individual, such as maturation of the immune system, intellectual development, and the onset of various pathologies. Whole-genome sequence-based metagenomics analyses and 16S rRNA gene sequencing have made it possible to define richness and diversity of bacterial species, and several computational tools have been used to describe and to compare microbial communities [8,9,10,11]. Ley et al. demonstrated that mammalian gut microbiota is markedly different from other free-living communities not associated with animal body habitats, suggesting a strong selective effect on the structure of the microbiota of mammals [12]. In humans, the dominant phyla are *Bacteroidetes*, *Firmicutes*, *Actinobacteria*, *Proteobacteria, Fusobacteria*, and *Verrucomicrobia*, with the two phyla *Firmicutes* and *Bacteroidetes* representing 90% of the gut microbiota [2,13]. To the phylum of the *Firmicutes* belong more than 200 different genera, including *Lactobacillus*, *Bacillus*, *Clostridium*, *Enterococcus*, and *Ruminicoccus*, to that of *Actinobacteria* the genera of *Bifidobacteria.* Meanwhile, to the phylum of *Proteobacteria* belongs *Enterobacteria* (*Escherichia coli* and other related species). Gut microbiota varies according to the intestine anatomical regions. In healthy adult subjects, the oesophagus presents only slight bacterial contamination as well as the stomach which is practically sterile when the pH is <3.0. However, in some situations, as for example, with the now very frequent use of proton pump inhibitors drugs, there is the proliferation of *Helicobacter Pylori*, *Enterococcus*, *Streptococcus*, *Staphylococcus,* and the potentially pathogenic *Escherichia coli* [13]. In the duodenum, the bacterial concentration is maintained at low levels by the bactericidal action of the bile. In about 50% of fasting cases, this concentration may slightly decrease and the flora consists largely of *E. coli* and other *Enterobacteria*, *Enterococci*, *Streptococci*, *Staphylococci*, *Lactobacilli*, and traces of *Bifidobacteria*. In the ileum, the concentration rises; *Lactobacilli* and *Bifidobacteria* are the most prevalent and *Bacteroides* and *Clostridia* appear. The large intestine, characterized by slow flow rates and neutral to mildly acidic pH, harbors by far the largest microbial community that is dominated by obligate anaerobic bacteria [3]. Although the mature microbiota is quite resilient, its richness and composition may vary in physiological conditions, in response to both internal and external challenges and according to age, ethnicity, lifestyle, drug use, and dietary regime, but also in a series of intestinal and extra-intestinal diseases [14,15,16]. 

Despite the variability in gut microbiota compositions, functional gene profiles are quite similar in different individuals, as highlighted by a series of studies involving very large populations [5,15]. As reported by Lozupone et al., core functions of the gut microbiota include central metabolic pathways and pathways involved in important gut functions, including carbohydrate and amino acid metabolism. Not all pathways are represented in the core and grouping genes into broad functional categories can mask meaningful interindividual differences in the function that occur at finer scales. Variable functions restricted to species or strain, including pathogenicity islands, vitamin and drug catabolism, motility and nutrient transporters, are intriguing targets for personalized diets and therapeutic strategies. Many genes are expressed only under specific conditions. For example, genes involved in carbohydrate metabolism and energy production are expressed at higher levels than predicted from metagenome data, underscoring that these processes are important in the gut [17].

In Western countries, in the last twenty years, the attention of researches has focused not on pathogenic bacteria, but on the microbiota of apparently healthy subjects, being by now clear that the correct functioning of the intestinal system, the removal of toxic substances, and the correct nutrient utilization depend on the intestinal microbiota. What is more, the existence of an intestine–brain axis determines the well-being of the organism in its entirety [18].

## 2. The Impact of Diet on the Human Gut Microbiota

Nutrition exerts short- and long-term influence on the microbial community with profound effects on human health. In fact, dietary-induced changes to microbiota have been progressively associated not only to host physiology but also to chronic illness, including obesity, immune, metabolic, and inflammatory bowel diseases [19,20,21]. As highlighted by Zmora et al., the above changes could partly explain and predict the interindividual variability in diet response observed in apparently similar conditions [22]. Currently, diet-microbiota interaction is beginning to be considered to set personalized nutritional regimes to deal with and to prevent some disorders or, more simply, to grant a healthy life [23].

The human interaction diet–microbiota originates at birth when sialylated human milk oligosaccharides (HMOs) are administered to infants [24]. It has been found that *Enterobacteria* appear in the first months of life (3–14 months), more precociously in artificially suckled infants, and remain present until the end of the 3rd–4th year [25]. Similarly, *Bifidobacterium* spp are more abundant in the gut microbiota of children than in that of adults and may gradually decrease through adulthood [26]. In Africa, where children are often precociously weaned, their microbiota is quite similar to that of adults, whose diet is notoriously poor in animal protein. During the first few years, gut microbiota influences the maturation of the immune system, nutrient absorption, and metabolism, and prevents pathogen colonization [26]. Throughout a lifetime, the richness of microbiota is substantially increased by the introduction of solid foods, while, on the contrary, it declines in aging, mainly in subjects in long-stay care with pronounced frailty and co-morbidities, likely due to reduced diet diversity. In addition, among the elderly, greater variability in the microbiota composition was observed, making it an effective biomarker of aging [27,28,29]. 

Nutrients can directly affect the gut microbiota by promoting or inhibiting the growth of microorganisms, indirectly influencing the host’s metabolism and its immune system or passively joining certain food-derived members to the microbiota, such as *Candida*, *Penicillium* fungi, and lactic-acid producing bacteria [30,31]. It was reported that macronutrient intake could shift the structure of microbiota within a day, thus demonstrating that it is able to respond very quickly to dietary intervention. However, this response has sometimes appeared transient since dietary-induced microbial changes disappear immediately after cessation of diet administration [21,32,33]. In this context, not only dietary timing and components but also the circadian oscillation of the gut microbiome and eating habits play a fundamental role [34,35,36,37]. In ad libitum high-fat feeding experiments, mice lack daily fluctuation in the relative abundances of *Bacteroidetes* and *Firmicutes* and the access to a high-fat diet in the dark phase only partially restores microbial oscillation (34,36). Moreover, the feeding restriction to either the light phase or the dark phase rescues the loss of fluctuations in the gut microbial composition of Per1 and Per2 knockout mice, thus reflecting the time-dependent metabolic profile and timed availability of nutrients for bacteria [38]. Several studies have reported that routine diets have a greater influence on gut microbiota than acute dietary strategies. An effective example comes from the differences in microbiota composition between African children, originating from a rural village of Burkina Faso where a dietary high in fiber content is prevalent, and urban European children, who eat Western-style diets rich in animal protein/fat. Africans showed enrichment in *Bacteroidetes* and depletion in *Firmicutes*, with a unique abundance of bacteria from the genus *Prevotella* and *Xylanibacter*, containing genes for cellulose and xylan hydrolysis, completely lacking in Europeans. In addition, *Enterobacteriaceae* (*Shigella* and *Escherichia*) were significantly under-represented in Africans. This evidence suggests that gut microbiota coevolved with the polysaccharide-rich diet of Africans, allowing them to maximize energy intake from fiber [39]. 

Structural and cohort studies have also reported an influence of diet on microbiota by geographic localization, urbanization, and seasonal cycle [40,41]. According to microbiota composition, healthy adults from nine provinces of China were clustered in relation to geography and ethnicity [42]. Vangay et al. demonstrated that emigrants from a non-Western country to the United States exhibited loss of gut microbiome diversity and function in which the strains USA-associated replace the native ones. These effects increase with the duration of the residence, in obesity condition, and across generations [43]. Urbanization mostly determines the loss of microbiota diversity and of particular bacterial species, such as *Treponema*, while a rural diet is associated with an increase in *Bacteroidetes* that allows populations to maximize energy intake from fiber [44]. Hutterites, an isolated population living in North America, eat frozen or canned food during winter and fresh vegetables and fruit during summer in which an increase and depletion of *Bacteroides* and *Actinobacteria,* respectively, were observed in their gut microbiota [39,45]. 

A wide variety of nutritional studies reported the impact on gut microbiota composition and function of micro- and macro-nutrients, such as carbohydrates (CHO), novel food components, food additives as well as of diets containing high fat/low fiber, low fat/high fiber, high- or low-proteins, fruits, and vegetables [20,21,22,23,46]. CHO fermentation is the preferred energy source for the gut microbiota and the amount of dietary CHO depends on many factors, such as meal size, chemical structure, food matrix, and preparation method, food form, rate of digestion, gut transit, and presence of enzyme inhibitors (e.g., tannins) [47]. Certain bacterial species are associated with CHO consumption, as demonstrated by the fact that its decline results in a progressive reduction in *Bifidobacterium* in obese subjects [48]. Vitamin D intake in humans was associated with decreased levels of circulatory bacterial lipopolysaccharide (LSP) and *Coprococcus* and *Bifidobacterium* abundance and increased abundance of *Prevotella* [22,49]. Omega-3 polyunsaturated fatty acids (PUFA) supplementation induces a decrease in *Faecalibacterium*, often associated with an increase in the *Bacteroidetes* and butyrate-producing bacteria belonging to the *Lachnospiraceae* family, as well as an increase in the production of anti-inflammatory compounds, such as short-chain fatty acids (SCFAs) [50]. Dietary proteins serve as the major source of nitrogen for colonic microbial growth and are essential to bacterial assimilation of carbohydrates and production of beneficial products including SCFAs. A ketogenic diet modifies bacteria taxa, richness, and diversity, thus exerting neuroprotective effects, influencing weight loss, enhancing longevity, and reducing the onset of different diseases [51]. A high-fat diet in healthy subjects is associated with increased and decreased levels of *Alistipes* and *Bacteroidetes* and *Faecalibacterium*, respectively, all changes associated with cardiovascular and metabolic diseases [23]. The Mediterranean diet is predominantly associated with a low ratio of *Firmicutes:Bacteroidetes* and with high production of SCFAs [52]. Vegans and vegetarians have significantly higher counts of certain *Bacteroidetes*-related taxonomic units compared to omnivores [53]. Fiber most consistently increases lactic acid bacteria, such as *Ruminococcus*, *E. rectale*, and *Roseburia*, and reduce *Clostridium* and *Enterococcus* species. In particular, a whole-grain wheat-based diet for 12-weeks was found to increase fasting plasma propionate that was associated with lower postprandial insulin concentrations [54]. Several studies demonstrated an increase in microbiota diversity and/or abundance following intact cereal fiber consumption, with effects from 24 h to 52 weeks [55,56]. Recently, Si et al. demonstrated that, in obese rats, gamma-aminobutyric acid (GABA) enriched rice bran (ERB) supplementation attenuated body weight gain and insulin resistance induced by a high-fat diet as well as energy metabolism abnormity. An ERB-containing diet stimulated butyrate and propionate production by promoting *Anaerostipes*, *Anaerostipes sp*. [57]. Polyphenols, found largely in the fruits, vegetables, cereals, increase *Bifidobacterium* and *Lactobacillus*, thus providing anti-pathogenic and anti-inflammatory effects and cardiovascular protection [22,23,58]. Finally, junk foods, or in any case, industrially produced foods, rich in preservatives, additives, sugars, and salt, but poor in soluble and insoluble fiber, negatively affect the microbiota [22,59,60]. 

## 3. Gut Microbiota Metabolites

Gut microbiota produces dietary-dependent, such as SCFAs, amino acids, lipids, vitamins, and dietary-independent products, such as LPS, peptidoglycan, neurotransmitters, and hormones [61,62,63,64]. Catabolism of resistant starch and anaerobic fermentation of dietary non-digestible food components contribute to the production of SCFAs. [65]. In healthy individuals, their amount and types are largely correlated to substrate availability, mostly polysaccharides and oligosaccharides, microbiota composition and intestinal transit time [66,67]. The most abundant SCFAs are acetate and propionate that are mainly produced by *Bacteroidetes*, whereas propionate is also produced by *Veilonella*, *Roseburia*, and *Ruminococcus*. The third important SCFA is butyrate, mainly formed by the phylum *Firmicutes* [66,67]. Propionate and butyrate can also be derived from peptide and amino acid fermentation, although the numbers of amino acid-fermenting bacteria have been estimated to constitute less than 1% of the large intestinal microbiota [68]. Acetate and propionate are absorbed by colonocytes via passive diffusion, electroneutral, or electrogenic uptake and pass through them into the portal vein and are thus transported in peripheral tissues [69]. On the contrary, butyrate is well known to act in host cells. Germ-free mice exhibited low, but measurable, concentrations of SCFAs in intestinal content compared to conventional mice [70]. Rats fed a diet rich in fiber show an increased SCFA concentration that increases further by replacing 10% of dietary wheat starch with inulin and decreases when fiber content was increased by 20% inulin. Moreover, the intake of inulin shifted the production of SCFAs from acetate to propionate and butyrate. In humans, it was observed that SCFA concentration in feces, in which acetate is predominant, does not reflect their rate of production in the intestine since most of them are absorbed by the host [71]. In addition to the compounds mentioned above, derivative aromatic SCFAs, including phenylacetate and phenylbutyrate, are obtained by the transformation of polyphenols or by the metabolism of microbial species in the gut, such as *Bacteroides*, *Clostridium*, *Eubacterium limosum*, and *Eggerthella lenta*. 

SCFAs are important energy sources for both gut microbiota and host intestinal epithelial cells. These metabolites exert diverse regulatory functions, and their effects on host physiology, metabolism regulation, inflammation, and immunity have been progressively documented [67,72]. SCFAs are inhibitors of histone deacetylases (described in detail later) and specific ligands for G protein-coupled receptors (GPCRs), such as GPR41 (FFAR3), GPR43 (FFAR2), and GPR109a (HCAR2), located on the surface of epithelial and immune cells [73]. They act as signaling molecules that influence the expansion and function of hematopoietic and non-hematopoietic cell lineages, the neutrophil chemotaxis, the maturation and function of microglia, and the maintenance of microglia homeostasis [74,75,76]. SCFAs induce reactive oxygen species (ROS), activate inflammasome assembly, thus increasing production of the downstream inflammatory cytokine IL-18, alter gut integrity, change cell proliferation, and exert anti-inflammatory, antitumorigenic, and antimicrobial effects [72,77,78]. Recently, the olfactory receptor 78 (Olfr78), a chemosensory found in vascular resistance beds in a variety of tissues, has been identified as a novel ligand for SCFAs, thus suggesting that it may regulate blood pressure by responding to gut microbiota. More specifically, this regulation might act as a mechanism to facilitate absorption after a meal [79]. SCFAs are essential for the maintenance of mucosal immunity by stimulating the expression of mucin (MUC2) synthesis and its secretion by goblet-like colon cells [80]. They also stimulate antimicrobial peptides (AMPs), so that they exert an innate defence against pathogens and modulate tight junction (TJs) formation and their permeability potentially through activation of Adenosine monophosphate-activated protein kinase (AMPK) [81,82]. SCFAs stimulate PPARγ and inhibit NF-κ, so they play a role in the regulation of cholesterol, lipid, glucose metabolism, and inflammatory response [83,84]. Additional transcription factors affected by them include Specificity Protein 1 (SP1), Activator Protein 1 (AP-1), Aryl hydrocarbon Receptor (AhR), and Forkhead box P3 (FOXP3). Lastly, SCFAs also carry out their activity into the mitochondria in which they are transferred by permeating the inner mitochondrial membrane in the non-esterified form and, then, are activated to Acyl-CoA thioesters and metabolized in the mitochondrial matrix [85]. SCFAs modulate tissue metabolism of carbohydrates and lipids, as demonstrated by an inhibitory effect on glycolysis and stimulation of lipogenesis or gluconeogenesis. SCFAs also modulate mitochondrial energy production by providing reducing equivalents to the respiratory chain and partly decreasing the efficacy of oxidative Adenosine triphosphate (ATP) synthesis [85].

Gut microbiota also synthesizes important compounds, such as methyl or acetyl groups, B vitamins, including riboflavin (B2), niacin (B3), biotin (B8), pantothenic acid (B5), and folate (B6), as well as various enzymes (methyltransferases, acetyltransferase, deacetylase, BirA ligase, phosphotransferases) that, as will be discussed later, play a role in DNA methylation and histone modifications, thus influencing all physiologic and pathologic processes in which epigenetic changes are fundamental [86]. It also synthesizes polyamines, low-molecular-weight aliphatic polycations, such as putrescine, spermidine, and spermine [87]. They are essential for the survival and virulence of several bacterial pathogens, such as *Helicobacter pylori*, *Shigella* spp, *Staphylococcus aureus,* and modulate systemic and mucosal adaptive immunity. What is more, their concentration is associated with cell growth defects, toxic effects, and carcinogenesis. In addition, polyamines regulate the integrity of the intestinal epithelial cells by stimulating the formation of intercellular junction proteins. 

Gut microbiota also produces a wide range of mammalian neurotransmitters, including dopamine, norepinephrine, serotonin, or gamma-aminobutyric acid (GABA). Dietary interventions are well known for their ability to alter the composition and function of the gut microbiome through the increase in GABA [88]. Pieces of evidence suggest that manipulation of these neurotransmitters by bacteria may have an impact in host physiology, and preliminary human studies are showing that microbiota-based interventions can also alter neurotransmitter levels.

## 4. Impact of Diet-Microbiota Interaction on Host Epigenome in Health and Disease

### 4.1. Diet, Microbiota Metabolites, and DNA Methylation

Epigenetics is the study of mitotically and meiotically hereditable changes that may influence gene expression without altering the DNA sequence [89,90]. DNA methylation and modifications of histone proteins are the most intensively studied among the major epigenetic modifications. 

The gut microbiota acts as a hub in integrating dietary signals with a multitude of host responses, thus originating complex crosstalk that is fundamental in achieving and maintaining homeostasis with the host. Molecular mechanisms underlying this crosstalk remain largely unknown, although recent evidence has suggested that epigenetic modifications are the means by which the nutritional profile, and the consequent gut microbiota structure, may impact the host both in physiological and pathological conditions according to the so-called “microbiota–nutrient metabolism–host epigenetics axis” [91,92,93,94,95,96,97]. 

**Figure 1:**
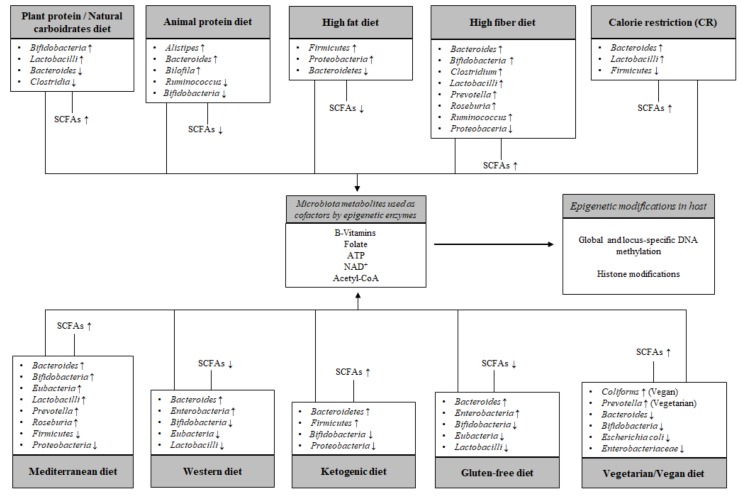
Schematic representation of the interaction between dietary patterns and gut microbiota on the host epigenetic modifications. Differences in exposure to diets influence the microbiota structure, the overall production of short-chain fatty acids (SCFAs), the most prominent end-products of microbial fermentation, and a series of dietary energy metabolites which are used as cofactors by epigenetic enzymes. Up-arrows indicate increase, down-arrows decrease.

In Figure 1, the impact of the most common dietary patterns on the gut microbiota composition and its metabolites, which have consequences on the host epigenome, is summarized.

In this context, a pilot study carried out in pregnant women revealed a strong association between bacterial predominance and epigenetic profiles. More specifically, in mothers in which *Firmicutes* were dominant, methylation profile analysis carried out in blood samples, revealed 568 hyper-methylated, including USF1, ACOT7, TAC1, LMNA, and SCD5, and 254 hypo-methylated genes, including FOXD1, KCNIP4, SERINC3, and MEF2A, some of which associated with the risk of cardiovascular disease (409 altered genes), lipid metabolism (72 altered genes), obesity (23 altered genes), and inflammatory response (85 altered genes) [98]. Recently, analyses of gut microbiota composition in DNA stool and genome-wide DNA methylation profiling in blood and visceral adipose tissue from obese subjects revealed a fully different DNA methylome pattern in both blood and adipose tissue in the group with low *Bacteroidetes: Firmicutes* ratio versus the group with high *Bacteroidetes:Firmicutes*. Two hundred fifty-eight genes were observed differentially methylated, such as HDAC7 and IGF2BP2, both implicated in glucose and energy homeostasis [99].

As highlighted by Cortese et al., epigenetics changes in the host may be induced by the microbiota through (i) alterations in the availability of chemical donors for DNA methylation or histone modifications, which depend on nutrition and the metabolic activities of the microbiota; (ii) DNA modifications caused by the mechanisms triggered by the incorporation of foreign genetic material into the genome; (iii) direct interaction with enzymes responsible for DNA methylation or histone modifications [100].

It is well known that most of the key molecules involved in one-carbon metabolism, including folic acid and vitamins B2, B12, and B6, are dietary- and microbiota-dependent, being susceptible to dietary intervention and gut dysbiosis [101]. They play a fundamental role in generating S-Adenosylmethionine (SAM), the main methyl-donating substrate for DNA Methyltransferases (DNMTs) and Histone Methyltransferases (HMTs). Microbiota enzymes that are exhausted in different human conditions, such as obesity, are recurrently involved in cofactor and vitamin metabolism [102]. Next to the direct introduction with the diet, folic acid is also synthesized from pteridine precursors (DHPP) and p-aminobenzoic acid (pABA) by specific bacteria constituents of the gut microbiota. Among these, many commensal *Lactobacillus* and *Bifidobacterium* species have been demonstrated to be important in folate production, so that they have attracted high consideration as probiotics [103]. It is plausible that the colon represents a local and substantial folate depot that participates in metabolism and contributes to circulating folate levels [103]. The capacity of folate biosynthesis most likely depends on intestinal microbiota structure, and variates within and between individuals, as a consequence of nutritional regime, use of antibiotics, and other lifestyle or medical factors. According to Schaible et al., maternal supplementation in mice of methyl-donors led to altered microbiota composition and colitis susceptibility in the offspring. What is more, at the level of the colonic mucosa, altered DNA methylation and expression of genes implicated in intestinal inflammation were observed [104]. Dietary methionine shapes both the host–microbiota structure and bacterial metabolism to generate substrates for SAM synthesis. *Escherichia coli* deficiency for an external source of methionine reduced in the host, SAM and phosphatidylcholine synthesis. The latter can activate the nuclear receptor NR5A, which suppresses grl-21 expression and, thus, inhibits mitochondrial fission and host lipid accumulation [105]. A well-defined role has also been attributed to intermediates of the tricarboxylic acid cycle (TCA), which is influenced by Acetyl-CoA produced by SCFAs. Among these intermediates, alpha-Ketoglutarate acts as co-substrate of Ten-eleven Translocation (TET) dioxygenases in charge of demethylation processes, meanwhile fumarate and succinate inhibit TET enzymes with consequently increased levels of DNA methylation [97]. 

A putative link between microbiota SCFAs production and epigenetic regulation has been investigated in obese and type 2 diabetes patients. The diversity of the gut microbiota, especially as regards the component producing butyrate, and the methylation levels of the FFAR3 promoter gene were significantly lower in the obese and type 2 diabetic patients compared to lean individuals, demonstrating a correlation between a higher body mass index and lower methylation of FFAR3 [106]. Altered microbiota and SCFA deficiency are also primary causal factors triggering type 1 diabetes (T1D). In fact, a diet rich in acetate and butyrate protected mice against T1D and this SCFA-induced protection happened via changes in gut/immune regulation, expanding T_reg_ cells and reducing pathogenic B cells, CD4+, and CD8+ T cells [107]. Butyrate impairs progression of nephropathy through, at least in part, protective effects on podocytes mediated by DNA methylation and Histone Deacetylases (HDACs) -dependent mechanisms, involving genes essential for podocyte function, as well as activation of GPR109a [108]. Donohoe et al. demonstrated that, in gnotobiotic mice colonized with wild-type or mutant strains of butyrate-producing *Butyrivibrio fibrisolvens*, a fiber-rich diet induced strong tumor-suppressive effects in a microbiota- and butyrate-dependent manner. What is more, butyrate was metabolized less in tumors where it accumulated, due to the Warburg effect, and exerted its histone deacetylase inhibitor function, thus stimulating histone acetylation and affecting apoptosis and cell proliferation [109]. 

A whole series of dietary bioactive compounds from various sources, which target enzymes involved in epigenetic gene regulation, are metabolized by the gut microbiota [96]. The microbial degradation of catechins, of which Epigallocatechin-3-Gallate (EGCG) is the major, by cleavage of the *O*-heterocycle and dihydroxylation produces phenolic acids that inhibit DNMTs’ activity by 60%–80% [110]. Remely et al. demonstrated that supplementation of EGCG induced a reduction in the *Firmicutes:Bacteroidetes* ratio and tissue-specific expression patterns as demonstrated by a decrease in mRNA levels of inflammatory IL-6, in the colon, MLH1 and DNMT1 in the liver, and an increase in DNMT1 in the colon. What is more, the hypermethylation of promoter region of MLH1 and DNMT1 was observed in the liver [111]. Black raspberries increase butyrate-producing bacteria such as *Anaerostipes*, and anti-inflammatory bacteria, such as *Akkermansia* and *Desulfovibrio*, and their microbiota metabolism results in reducing the expression of DNMT1 and promoter methylation levels of genes involved in the WNT-signalling pathway in tumor tissues with consequently reduced expression of proteins including β-catenin, E-cadherin, and MKI67 [112]. Several cruciferous vegetables contain glucosinolate of sulforane (SFN) that is able to downregulate DNMTs, thus leading to demethylation of specific CpG sites located within CpG islands of the telomerase reverse transcriptase (TERT) gene and a reduction in its expression in cancer [96,113]. 

A series of studies demonstrated that the microbiota affects the methylation status of epithelial cells, thus playing a functional role. In colonic intestinal epithelial cells from germ-free mice, DNA was observed markedly hypermethylated and this was associated with a strong loss of ten-eleven-translocation activity, lower DNA methyltransferase activity, and lower circulating levels of the 1-carbon metabolite, folate [114]. Besides, an enrichment for differentially methylated regions adjacent to genes regulating the cytotoxicity of NK cells and, in particular, to those regulating the cross-talk between NK cells and target cells, was found [115]. Microbiota which dynamically influences the postnatal development of the transcriptome in epithelial cells and targets only a subset of responsive genes through their DNA methylation status, included those involved in immune pathways and metabolic processes [116]. What is more, it was observed that the methylation level of TLR4 promoter gene (Toll-like receptor 4), a member of the toll-like receptor family that directs intestinal barrier function and immune-regulatory responses, was significantly lower in the intestinal epithelial cells of the large intestine of germ-free mice than in those of conventional mice. This leads to the consideration of the role of microbiota in the regulation of intestinal inflammation by contributing to the maintenance of intestinal symbiosis [91]. In addition, TLR2 knockout mice are characterized by a methylation level increase in Anpep and Ift2, which are two genes functionally correlated to the immune process [117]. More recently, influences of colonizing microbiota pathogenic bacteria, such as *Helicobacter pylori* and *Klebsiella* spp, on the host methylation patterns have been reported. Individuals with a *Helicobacter pylori* infection displayed very high methylation levels in eight regions of CpG islands (p16 core region, p16 noncore region, LOX, THBD, FLNc, HRASLS, HAND1, and p41ARC) in the gastric mucosae, thus indicating that the infection may induce DNA methylation [118].

### 4.2. Diet, Microbiota Metabolites, and Histone Modifications

Alteration of chromatin state is a possible mechanism by which gut microbiota mediates the dietary effects on the host. The majority of histone modifications are reversible and responsive to metabolic changes. Direct evidence has demonstrated that microbiota metabolites impact host histone modifications in a diet-dependent manner in several tissues, including those outside the alimentary tract [92,93,94,95]. Globally, SCFAs and a series of metabolites and cofactors, such as SAM, Acetyl-CoA, ATP, Fe^2+^/Fe^3+^, and alpha-Ketoglutarate, inhibit or promote histone modifications [97,119]. The epigenetic role of SCFAs as inhibitors of Histone Deacetylases (HDACs), with butyrate>propionate>acetate, that cause increased levels of histone acetylation, decondensation, and relaxation of chromatin, has been largely demonstrated [68]. SCFAs exert anti-inflammatory effects in hematopoietic cells by stimulating histone acetylation, increasing expression of the FOXP3 gene in CD4^+^ T cells and promoting the differentiation of T_reg_ cells, namely specialized subpopulation of T cells that suppress the immune response, thereby maintaining homeostasis and self-tolerance [120,121]. The HDAC inhibition by SCFAs promotes the increase in the expression of β-defensin 2 and β-defensin 3, thus ultimately leading to protection against severe infections [122]. Butyrate inhibits HDAC1 and HDAC2 in contrast to acetate and propionate that specifically inhibit HDAC2 and HDAC3 [123]. To this purpose, it was demonstrated that the action of butyrate is often mediated through Sp1/Sp3 binding sites whose HDAC activity leads to histone hyperacetylation and transcriptional activation of the p21(Waf1/Cip1) gene involved in the arrest of cell cycling [123]. Butyrate, acting as a HDAC inhibitor, suppresses NF-kB and STAT1 activation, inhibits interferon γ production, and upregulates peroxisome proliferator-activated receptor γ (PPARγ), thus downstream inducing the production of pro-inflammatory cytokines (IL-1B, IL-8) [98,124]. Nancey et al. observed that butyrate in macrophages increased global acetylation and decreased the expression of LPS-induced pro-inflammatory cytokines IL-6 and IL-12 [125]. What is more, it de-represses silenced genes in cancer cells, such as cell cycle inhibitor p21 and the proapoptotic protein Bcl-2 homologous antagonist/killer (BAK) and activates these genes in normal cells [126]. Treatment of cells with butyrate, acetate, propionate, and valerate results in the increase in histone H3 and H4 acetylation that induces cell cycle arrest and expression of cellular differentiation markers as well as the downregulation of neuropilin-1 (NRP1), an effective apoptotic and angiogenesis regulator [127]. If in cancer cells the Warburg effect leads to an intracellular accumulation of butyrate, with consequent blocks of proliferation, in normal colonocytes butyrate promotes their proliferation by acting as a carbon donor for Acetyl-CoA and histone acetylation. In these conditions, low amounts of butyrate favor HAT activity and proliferation at the bottom of the cript. Meanwhile, high levels determine HDAC inhibition and apoptosis at the top [76,128]. 

Recently it was reported that *Lactobacillus reuteri* 6475 produces a novel form of folate that is analogous to 5,10-methenyl-THF but incorporates an additional methyl group at the folate’s reactive center, thus originating the 5,10-methylmethenyl-THF polyglutamate or 5,10-ethenyltetrahydrofolyl (5,10-EtTHF) polyglutamate. EtTHF is biologically active and can transfer two carbons onto homocysteine, resulting in the formation of the amino acid ethionine that is an analog to methionine. Interestingly, ethionine activity was found associated with the suppression of histone methylation and ethylation of histone lysine residues [129].

Kuang et al. reported in mice that intestinal microbiota programs diurnal metabolic rhythms through HDAC3 that was recruited rhythmically to chromatin, and produced synchronized diurnal oscillations in histone acetylation, metabolic gene expression, and nutrient uptake. HDAC3 also co-activated Estrogen-Related Receptor α (ERRα), inducing microbiota-dependent rhythmic transcription of the lipid transporter gene Cd36 and promoting lipid absorption and dietary-induced obesity [130].

The introduction of gut microbiota in germ-free mice modulates the histone code in a manner dependent on the bacteria composition. In colon, liver, and adipose tissues from these mice, increased levels of H3K27me3 and H3K36 and decreased levels of H3K18me1, H3K23, K27me2, and K36me1 were observed. Contrarily, other modifications display tissue-specificity, such as H3K27me1 and H3K36me2 which increased in adipose tissues, meanwhile, decreased in liver and colon. Colonization of germ-free mice has demonstrated the capacity of microbiota-specific species to activate Major Histocompatibility Complex (MHC) class II genes, probably by regulating MHC gene expression through DNA methylation and histone acetylation and methylation [97,131].

The nuclear SIRT1, a sirtuin deacetylase that plays a vital role in the regulation of mitochondrial biogenesis, metabolism, stress responses, genome stability, and ultimately aging, is regulated by SCFAs [132]. In addition, resveratrol, which has been shown to have beneficial effects on metabolic syndrome-related alterations, is able to significantly increase SIRT1 activity [133]. It has been postulated that the health benefits of resveratrol are largely dependent on the gut microbiota that actively participates in its metabolism by increasing its availability from precursors and producing resveratrol derivatives. *Bifidobacteria infantis* and *Lactobacillus acidophilus* are responsible for resveratrol production from piceid. Meanwhile, *Slackia equolifaciens* and *Adlercreutzia equolifaciens* produce dihydropiceid and dihydroresveratrol [134]. 

Lastly, the foodborne Gram-positive facultative anaerobic-bacterium *Listeria monocytogenes* was found to induce globally acetylation of histone H4 (lysine 8) and phosphorylation/acetylation of histone H3 (serine 10/lysine 14) and at the *il8* promoter in Human Umbilical Vein Endothelial Cells (HUVEC) cells as well as recruitment of the histone acetylase Cyclic Adenosine 3′,5′-Monophosphate Response Element Binding Protein (CREB) binding protein, thus contributing to cytokine expression of human endothelial cells [135]. Similarly, *Helicobacter pylori* increased the expression of p21(WAF1) that was associated with the release of HDAC-1 from the p21(WAF1) promoter and hyper-acetylation of histone H4. This evidence can contribute to dissecting the molecular mechanisms underlying the development and progression of *H. pylori*-associated diseases [136]. 

In Table 1, putative interactions between gut microbial metabolites and host epigenetic changes, as well as their potential involvement in health and diseases, are detailed.

Therapeutic application in clinical practice based on diet–microbiota–host epigenome interaction is progressively attracting interest. The involvement of this interaction in different diseases, such as cancer, inflammatory bowel disease, obesity, and metabolic syndrome, represents the basis for both determining and understanding how the microbiome is able to influence human health and disease progression and developing novel approaches in their diagnosis and treatment. Many examples can be reported about the role of dietary foods and additives containing probiotics or metabiotics in supporting human health and in treating different diseases, which is reviewed in [143,144,145]. *Bifidobcaterium breve* and *Lactobacillus rhamnosus* GG exerted anti-inflammatory effects by inhibiting global DNA methylation and histone acetylation, thus reducing levels of the inflammatory factors IL-2, IL-7, and CD40. The probiotics *Akkermansia muciniphyla*, that exert a favorable effect on the disrupted metabolism associated with obesity, and *Faecalibacterium prausnitzii,* whose absence was associated with Crohn’s disease, upregulate HDAC3 and HDAC5, affect the expression of genes involved in host lipid metabolism and epigenetic activation or silencing of gene expression. Oral introduction of *Enterococcus faecalis* induced activation of 42 genes in the gut epithelial cells that are involved in the regulation of the cell cycle, cell death, and signaling. 

## 5. Conclusions

The relationship among human health, gut microbiota, and nutrition represents one of the most promising and challenging topics for researchers. Indeed, the microbiota is a dynamic community, undergoing changes in accordance with eating habits through the lifetime of individual hosts, and has great metabolic potential able to act on pharmacological targets and bioactive compounds. In this frame, although it is understood that epigenetic modifications activated in response to different dietary patterns appear to be fundamental in regulating the interaction between the gut microbiota and the host cells, the research on molecular and signaling pathways underlying such interaction still remains limited. What is more, valid and unique biomarkers of healthy microbiota have not yet been identified. Indeed, a globally accepted definition of a “health microbiota model” that connects all the information about microbiota structure, environmental factors, host diet, and metabolism is still missing. Moreover, the frame is made more complex by the interactions with the genetic interindividual variability of the hosts. Furthermore, it needs to be taken into account that in most studies on microbiota–host interactions murine models have become the organism of choice systematically. However, translating microbiota-related mouse data to humans requires some considerations. Despite important similarities, mice are not humans considering differences in the gut microbiota composition as well as in size, metabolic rate, and dietary habits as well as in anatomy, physiology, and genetics. In addition, the interaction between gut microbiota and the host is host-specific and so complex that evidence in mouse models should be applied with caution. It is likely that the limitations discussed above have significantly slowed down the development of targeted therapeutic approaches. The overcoming of these limitations could allow the identification of potential targets and the development of appropriate microbiota intervention strategies for improving human health. Future research should consider the effectiveness and application of several approaches, including the potential effects of prebiotic, probiotic, metabiotic, and fecal microbiota transplantation. However, although enticing, the promise of the above approaches is still limited by the literature gaps that have not yet deepened causal relationships between the gut microbiota and diseases at molecular levels and ascertained their safety since they might exert both beneficial and detrimental effects according different the clinical context and interindividual variations.

## Figures and Tables

**Table 1:** Gut microbiota metabolites, their biological function, and their implication in inducing epigenetic changes in the host.

Gut Microbiota Metabolites	Metabolite Producing Bacteria	Biological Functions of Metabolites	Metabolite-Induced Epigenetic Changes	Epigenetics-Associated Effects	Associated Diseases	Ref.
*Short-chain fatty acids (SCFAs)*: Acetate, propionate, butyrate, iso-butyrate, caproate, branched SCFAs (BCFAs), hexanoate, lactate, 2-methylpropionate, valerate, iso-valerate	*Lactobacillus, Eubacterium, Roseburia, Anaerostipes hadrus, Faecalibacterium, Coprococcus catus, Clostridia* (clusters IV and XIVa)	Regulation of: fatty acid, glucose, and cholesterol metabolism mucin synthesis synthesis of AMPs daily turnover of the epithelial lining and stem cell proliferation gut integrity by TJs neutrophil functions differentiation and function of Th1, Th7, and regulatory T (T_reg_) cells intestinal macrophage activation and recruitment dendritic cells in the induction of tolerance Suppression of pro-inflammatory cytokine secretion Improvement in insulin sensitivity and weight control Energy source for colonocytes	Inhibition of DNMT enzymes Decreased DNA methylation Inhibition of MBD2 Inhibition of HDACs Increased histone acetylation Activation of HAT Increased histone acetylation	Upregulation of FOXP3, β-defensin 2 and 3, ADIPOQ, RETN, Sp1/Sp3, BAK1, CDKN1A, CDKN1B, PPARγ, IFNγ, FAS, NOS2, CD36, IL-6, IL-8, IL-12B, ERRα, MHC class II, USF1, ACOT7, TAC1, LMNA, SCD5, HDAC7, IGF2BP, and SIRT1 genes LINE-1 DNA methylation Downregulation of NRP1, NF-kB, FTO, MC4R FOXD1, KCNIP4, SERINC3, MEF2A, and STAT1 genes	Inflammatory bowel disease, cardiovascular disease, ulcerative colitis, Crohn’s disease, obesity, metabolic syndrome, colorectal cancer, type 1 diabetes, type 2 diabetes, nephropathy, autism spectrum disorders	[65,66,67,68,69,70,71,72,73,74,75,76,77,78,79,80,81,82,83,84,85,91,96,97,98,99,106,107,108,109,117,119,120,121,122,123,124,125,126,127,128,130,131,132,133,134]
*Polyunsaturated fatty acid (PUFAs):* Arachidonic acid, docosahexaenoic acid, conjugated linoleic acids, conjugated linoleic acids, linoleic acid derivative	*Bifidobacterium, Roseburia, Lactobacillus, Klebsiella, Enterobacter, Citrobacter, Clostridium*	Maintenance of intestinal barrier function Regulation of intestinal IgA production Improvement in insulin sensitivity Regulation of development and function of the central nervous system	Inhibition of DNMTs activity Decreased DNA methylation Decreased histone methylation and phosphorylation Increased SIRT1 deacetylation activity	Dowregulation of EZH2 and CDK2 genes Upregulation of CDH1, PRKAA1, and IGFBP3 genes	Chronic systemic inflammation, hyperinsulinemia, depression, cognitive anxiety	[91,117]
*Vitamins:* Vitamin B1 (thiamine), vitamin B2 (riboflavin), vitamin B3 (niacin), vitamin B5 (pantothenic acid), vitamin B6 (pyridoxine), vitamin B8 (biotin), vitamin B9 (folate), vitamin B12 (cobalamin), vitamin K	*Bifidobacterium, Lactobacillus,* *Bacillus subtilis, Escherichia coli, Bacteroidetes, Fusobacteria, Proteobacteria, Actinobacteria*	Cofactors for enzymatic reactions Regulation of immune system, cell proliferation, and nutrient absorption Antioxidant properties Improvement in gut motility, blood glucose, lipid, and energy metabolism Protection against pathogens Prevention of allergies	Activation of DNMTs Increased DNA methylation Activation of HMTs and HATs Increased histone methylation and acetylation Activation of histone phosphorylation, biotinylation, and acylation Inhibition of Sirtuins (Class III HDAC)	Regulation of global DNA and histone methylation Transposable element repression	Crohn’s disease, type 2 diabetes, inflammatory bowel disease	[86,87,91,101,102,103,104,105,114,117,129]
*Bile salts:* cholate, hyocholate, deoxycholate, chenodeoxycholate, α-muricholate, β-muricholate, ω-muricholate, taurocholate, glycocholate, taurochenoxycholate	*Bifidobacterium, Lactobacillus,* *Bacteroides, Clostridium,* *Enterobacter, Eubacterium, Escherichia*	Absorption of dietary fats and lipid-soluble vitamins Regulation of bile acid, cholesterol, glucose, lipid, and energy homeostasis Maintenance of intestinal barrier function	Activation of HDACs Decreased histone acetylation		Colorectal cancer, Alzheimer’s disease, Parkinson’s disease, Huntington’s disease, infectious colitis	[137]
*Polyamines:* Putrescine, cadaverine, sperimidine, spermine	*Campilobacter jejuni, Clostridium saccharolyticum*	Promoting of cell growth, apoptosis and accumulation of Ca^2+^ in mitochondria Activation of production of mucus, secretory IgA, and intercellular junction proteins Maintenance of intestinal barrier function Regulation of immune system and mucosal adaptive immunity	Inhibition of DNMTs Decreased DNA methylation Inhibition of HDMs Increased histone methylation		Colorectal cancer, bacterial vaginosis	[138]
*Phenolic compounds:* hydroxicinnamic acids, hydroxycoumarins, benzoates, hydroxybenzaldehydes, hydroxyphenylacetic acids, hydroxyphenylpentanoic acids, p-cresol, hydroxyphenylpropanoic acids, valerolactones	*Lactobacillus, Bifidobacterium, Subdoligranulum, Clostridium difficile*	Promotion of anti-inflammatory and anti-oxidant response Regulation of gut metabolism and immunity Maintenance of intestinal barrier function Improvement in glucose uptake Inhibition platelet aggregation Estrogen-modulating effects	Inhibition of DNMTs Decreased DNA methylation Inhibition of HMTs and HATs Decreased histone methylation and acetylation Inhibition of HDACs Increased histone acetylation	Downregulation of MLH1, β-catenin, E-cadherin and MKI67 genes Upregulation of SIRT1 and Klotho gene	Colon cancer, metabolic syndrome	[96,110,111,112,139]
*Choline metabolites:* methylamine, dimethylamine, thrimethylamine, dimethylglicine, betaine	*Bifidobacterium, Firmicutes, proteobacteria, Actinobacteria*	Regulation of cell membrane function, methyl transfer reactions, and neurotransmission Modulation of lipid metabolism and glucose homeostasis Improvement in insulin sensitivity	Activation of DNMTs Increased DNA methylation	Global DNA methylation regulation	Cardiovascular disease, obesity, metabolic syndrome, type 2 diabetes, depression, anxiety, schizophrenia, Alzheimer’s disease	[104]
*Tryptophan metabolites:* indole, indole-3-acetate, indole-3-aldehyde, indole-3-carboxaldehyde, indole-3-propionate, kynurenine, quinolic acid, serotonin, tryptamine	*Clostridium sporogenes, Escherichia coli, Lactobacillus spp, Bifidobacterium Eubacterium hallii*	Maintenance of mucosal homeostasis and intestinal barrier function Production of antioxidant and neuro-protectant products Regulation of insulin responsiveness, energy expenditure and specialization of innate lymphoid cells	Activation of AhR Activation of DNMTs Increased DNA methylation	Regulation of genes containing XRE boxes Upregulation of FOXP3 Downregulation of IL-17	Inflammatory bowel disease, insulin resistance, depression, sleep disorder	[91,117,140]
*Aminoacids:* γ-aminobutyric acid (GABA), glycine, aspartic acid, glutamic acid, acetylcholine	*Clostridium, Lactobacillus, Bifidobacterium*	Regulation of immune system			Alzheimer’s disease, depression, anxiety	[57,88]
*Steroid hormones:* cortisol, androgens, estrogens	*Clostridium scindens,*	Regulation of weight gain and lipid deposition Influencing structure and levels of cortisol that is converted into androgens	Conversion of catechol estrogens into methoxy derivatives: 2-MeOE2 and 4-MeOE2 *via* COMT		Breast, ovarian and endometrial cancer	[141]
*Polysaccharide A, Polysaccharide B, Exopolysaccharides*	*Bacteroides fragilis, Lactobacillus*	Regulation of innate and adaptive immunity			Intestinal inflammation	[142]

2-MeOE2; 2-Methoxyestradiol; 4-MeOE2: 4-Methoxyestradiol; ACOT7; Acyl-CoA Thioesterase 7; ADIPOQ: Adiponectin; AhR: Aryl Hydrocarbon Receptor; AMPs: Antimicrobial Peptides; BAK1: BCL2 Antagonist/Killer 1; CD36: CD36 Molecule; CDH1: Cadherin 1; CDK2: Cyclin Dependent Kinase 2; CDKN1A: Cyclin Dependent Kinase Inhibitor 1A; CDKN1B: Cyclin Dependent Kinase Inhibitor 1B; COMT; Catechol-O-Methyltransferase; DNMT: DNA Methyltransferase; ERRa: Estrogen Related Receptor Alpha; EZH2: Signal Transducer and Activator of Transcription 1; FAS: Fas Cell Surface Death Receptor; FOXD1: Forkhead Box D1; FOXP3: Forkhead Box P3; FTO: FTO Alpha-Ketoglutarate Dependent Dioxygenase; HAT; Histone Acetylase; HDAC: Histone Deacetylase; HDAC7: Histone Deacetylase 7; HDM: Histone Demethylase; HMT: Histone Methyltransferase; IFNg: Interferon Gamma; IGF2BP2: Insulin Like Growth Factor 2 MRNA Binding Protein 2; IGFBP3: Insulin Like Growth Factor Binding Protein 3; IL-12B: Interleukin 12B; IL-17: Interleukin 17; IL-6: Interleukin 6; IL-8: Interleukin 8; KCNIP4: Potassium Voltage-Gated Channel Interacting Protein 4; LAMN: Lamin A/C; LINE-1: Long Interspersed Nuclear Element-1; MBD2: Methyl-Cpg-Binding Domain Protein 2; MC4R: Melanocortin 4 Receptor; MEF2A: Myocyte Enhancer Factor 2A; MHC Class II: Major Histocompatibility Complex, Class II; MKI67: Marker Of Proliferation Ki-67; MLH1: MutL Homolog 1; NF-kB: Nuclear Factor Kappa B; NOS2: Nitric Oxide Synthase 2; NRP1: Neuropilin 1; PPARg: Peroxisome Proliferator Activated Receptor Gamma; PRKAA1: Protein Kinase AMP-Activated Catalytic Subunit Alpha 1; RETN: Resistin; SCD5: Stearoyl-CoA Desaturase 5; SERINC3: Serine Incorporator 3; SIRT1: Sirtuin 1; Sp1/Sp3: Sp1 and Sp3 Transcription Factor; STAT1: Signal Transducer And Activator Of Transcription 1; TAC1: Tachykinin Precursor 1; TJs: Tight Junctions; USF1: Upstream Transcription Factor 1; XRE: Xenobiotic-Responsive Element.

## References

[1] Sommer F., Bäckhed F. (2013). The gut microbiota — masters of host development and physiology. Nat. Rev. Genet..

[2] Hollister E.B., Gao C., Versalovic J. (2014). Compositional and functional features of the gastrointestinal microbiome and their effects on human health. Gastroenterol..

[3] Thursby E., Juge N. (2017). Introduction to the human gut microbiota. Biochem. J..

[4] Qin J., Li R., Raes J., Arumugam M., Burgdorf K., Manichanh C., Nielsen T., Pons N., Levenez F., Yamada T. (2010). A human gut microbial gene catalogue established by metagenomic sequencing. Nature.

[5] Huttenhower C., Gevers D., Knight R., Abubucker S., Badger J.H., Chinwalla A.T., Creasy H.H., Earl A.M., Fitzgerald M.G., The Human Microbiome Project Consortium (2012). Structure, function and diversity of the healthy human microbiome. Nature.

[6] Ursell L.K., Metcalf J.L., Parfrey L.W., Knight R. (2012). Defining the human microbiome. Nutr. Rev..

[7] Heintz-Buschart A., Wilmes P. (2018). Human Gut Microbiome: Function Matters. Trends Microbiol..

[8] Andersson A.F., Lindberg M., Jakobsson H., Bäckhed F., Nyrén P., Engstrand L. (2008). Comparative Analysis of Human Gut Microbiota by Barcoded Pyrosequencing. PLOS ONE.

[9] Schloss P.D., Westcott S.L., Ryabin T., Hall J.R., Hartmann M., Hollister E.B., Lesniewski R.A., Oakley B.B., Parks N.H., Robinson C.J. (2009). Introducing mothur: Open-Source, Platform-Independent, Community-Supported Software for Describing and Comparing Microbial Communities. Appl. Environ. Microbiol..

[10] Hartman A.L., Riddle S., McPhillips T., Ludäscher B., Eisen J.A. (2010). Introducing W.A.T.E.R.S.: A Workflow for the Alignment, Taxonomy, and Ecology of Ribosomal Sequences. BMC Bioinform..

[11] Caporaso J.G., Kuczynski J., Stombaugh J., Bittinger K., Bushman F.D., Costello E.K., Fierer N., Peña A.G., Goodrich J.K., Gordon J.I. (2010). QIIME allows analysis of high-throughput community sequencing data. Nat. Methods.

[12] Ley R.E., Lozupone C.A., Hamady M., Knight R., Gordon J.I. (2008). Worlds within worlds: Evolution of the vertebrate gut microbiota. Nat. Rev. Genet..

[13] Rinninella E., Raoul P., Cintoni M., Franceschi F., Miggiano G.A.D., Gasbarrini A., Mele M.C. (2019). What is the Healthy Gut Microbiota Composition? A Changing Ecosystem across Age, Environment, Diet, and Diseases. Microorganisms.

[14] Sekirov I., Russell S.L., Antunes L.C.M., Finlay B.B. (2010). Gut Microbiota in Health and Disease. Physiol. Rev..

[15] Ehrlich S.D. (2010). The MetaHIT Consortium MetaHIT: The European Union Project on Metagenomics of the Human Intestinal Tract. Metagenomics of the Human Body.

[16] Le Chatelier E., Nielsen T., Qin J., Prifti E., Hildebrand F., Falony G., Almeida M., Arumugam M., Batto J.M., Kennedy S. (2013). Richness of human gut microbiota correlates with metabolic markers. Nature.

[17] Lozupone C.A., Jesse I., Stombaugh J.I., Gordon J.I., Jansson J.K., Knight R. (2012). Diversity, stability and resilience of the human gut microbiota. Nature.

[18] Osadchiy V., Martin C.R., Mayer E.A. (2019). The Gut–Brain Axis and the Microbiome: Mechanisms and Clinical Implications. Clin. Gastroenterol. Hepatol..

[19] Flint H.J., Duncan S.H., Scott K.P., Louis P. (2014). Links between diet, gut microbiota composition and gut metabolism. Proc. Nutr. Soc..

[20] Gentile C.L., Weir T.L. (2018). The gut microbiota at the intersection of diet and human health. Science.

[21] Leeming E., Johnson A.J., Spector T.D., Le Roy C.I., Le Roy C. (2019). Effect of Diet on the Gut Microbiota: Rethinking Intervention Duration. Nutrients.

[22] Zmora N., Suez J., Elinav E. (2018). You are what you eat: Diet, health and the gut microbiota. Nat. Rev. Gastroenterol. Hepatol..

[23] Kolodziejczyk A.A., Zheng D., Elinav E. (2019). Diet–microbiota interactions and personalized nutrition. Nat. Rev. Genet..

[24] Charbonneau M.R., O’Donnell D., Blanton L.V., Totten S.M., Davis J.C.C., Barratt M.J., Cheng J., Guruge J., Talcott M., Bain J. (2016). Sialylated Milk Oligosaccharides Promote Microbiota-Dependent Growth in Models of Infant Undernutrition. Cell.

[25] Milani C., Duranti S., Bottacini F., Casey E., Turroni F., Mahony J., Belzer C., Palacio S.D., Montes S.A., Mancabelli L. (2017). The First Microbial Colonizers of the Human Gut: Composition, Activities, and Health Implications of the Infant Gut Microbiota. Microbiol. Mol. Biol. Rev..

[26] Derrien M., Alvarez A.-S., De Vos W.M. (2019). The Gut Microbiota in the First Decade of Life. Trends Microbiol..

[27] Claesson M.J., Jeffery I.B., Conde S., Power S.E., O’Connor E.M., Cusack S., Harris H.M.B., Coakley M., Lakshminarayanan B., Sullivan O.O. (2012). Gut microbiota composition correlates with diet and health in the elderly. Nature.

[28] García-Peña C., Álvarez-Cisneros T., Quiroz-Baez R., Friedland R.P. (2017). Microbiota and Aging. A Review and Commentary. Arch. Med Res..

[29] Casati M., Ferri E., Azzolino D., Cesari M., Arosio B. (2019). Gut microbiota and physical frailty through the mediation of sarcopenia. Exp. Gerontol..

[30] A Segre J. (2015). Microbial growth dynamics and human disease. Science.

[31] David L., Materna A.C., Friedman J., Campos-Baptista M.I., Blackburn M.C., Perrotta A., Erdman S.E., Alm E.J. (2014). Host lifestyle affects human microbiota on daily timescales. Genome Biol..

[32] David L.A., Maurice C.F., Carmody R.N., Gootenberg D., Button J.E., Wolfe B.E., Ling A.V., Devlin A.S., Varma Y., Fischbach M.A. (2013). Diet rapidly and reproducibly alters the human gut microbiome. Nature.

[33] Zarrinpar A., Chaix A., Yooseph S., Panda S. (2014). Diet and feeding pattern affect the diurnal dynamics of the gut microbiome. Cell Metab..

[34] Thaiss C.A., Zeevi D., Levy M., Segal E., Elinav E. (2015). A day in the life of the meta-organism: Diurnal rhythms of the intestinal microbiome and its host. Gut Microbes.

[35] Liang X., Fitzgerald G.A. (2017). Timing the Microbes: The Circadian Rhythm of the Gut Microbiome. J. Biol. Rhythm..

[36] Kaczmarek J.L., Musaad S.M., Holscher H. (2017). Time of day and eating behaviors are associated with the composition and function of the human gastrointestinal microbiota. Am. J. Clin. Nutr..

[37] Kaczmarek J.L., Thompson S.V., Holscher H. (2017). Complex interactions of circadian rhythms, eating behaviors, and the gastrointestinal microbiota and their potential impact on health. Nutr. Rev..

[38] De Filippo C., Cavalieri D., Di Paola M., Ramazzotti M., Poullet J.B., Massart S., Collini S., Pieraccini G., Lionetti P. (2010). Impact of diet in shaping gut microbiota revealed by a comparative study in children from Europe and rural Africa. Proc. Natl. Acad. Sci. USA.

[39] Gupta V.K., Paul S., Dutta C. (2017). Geography, Ethnicity or Subsistence-Specific Variations in Human Microbiome Composition and Diversity. Front. Microbiol..

[40] Zuo T., Kamm M.A., Colombel J.-F., Ng S.C. (2018). Urbanization and the gut microbiota in health and inflammatory bowel disease. Nat. Rev. Gastroenterol. Hepatol..

[41] Fragiadakis G.K., Smits S.A., Sonnenburg E.D., Van Treuren W., Reid G., Knight R., Manjurano A., Changalucha J., Dominguez-Bello M.G., Leach J. (2018). Links between environment, diet, and the hunter-gatherer microbiome. Gut Microbes.

[42] Zhang J., Guo Z., Xue Z., Sun Z., Zhang M., Wang L., Wang G., Wang F., Xu J., Cao H. (2015). A phylo-functional core of gut microbiota in healthy young Chinese cohorts across lifestyles, geography and ethnicities. ISME J..

[43] Vangay P., Johnson A.J., Ward T.L., Al-Ghalith G.A., Shields-Cutler R.R., Hillmann B.M., Lucas S.K., Beura L.K., Thompson E.A., Till L.M. (2018). US Immigration Westernizes the Human Gut Microbiome. Cell.

[44] Emmanouil A., Bachar D., Yasir M., Musso D., Djossou F., Gaborit B., Brah S., Diallo A., Ndombe G.M., Mediannikov O. (2018). Treponema species enrich the gut microbiota of traditional rural populations but are absent from urban individuals. New Microbes New Infect..

[45] Davenport E., Mizrahi-Man O., Michelini K., Barreiro L.B., Ober C., Gilad Y. (2014). Seasonal Variation in Human Gut Microbiome Composition. PLoS ONE.

[46] Markowiak P., Śliżewska K. (2017). Effects of Probiotics, Prebiotics, and Synbiotics on Human Health. Nutrients.

[47] Wilson K., Situ C. (2017). Systematic Review on Effects of Diet on Gut Microbiota in Relation to Metabolic Syndromes. J. Clin. Nutr. Metab..

[48] Hold G., Schwiertz A., Aminov R., Blaut M., Flint H.J. (2003). Oligonucleotide Probes That Detect Quantitatively Significant Groups of Butyrate-Producing Bacteria in Human Feces. Appl. Environ. Microbiol..

[49] Luthold R.V., Fernandes G.D.R., De Moraes A., Folchetti L.G., Ferreira S.R. (2017). Gut microbiota interactions with the immunomodulatory role of vitamin D in normal individuals. Metabolism.

[50] Costantini L., Molinari R., Farinon B., Merendino N. (2017). Impact of Omega-3 Fatty Acids on the Gut Microbiota. Int. J. Mol. Sci..

[51] Paoli A., Mancin L., Bianco A., Thomas E., Mota J.F., Piccini F. (2019). Ketogenic Diet and Microbiota: Friends or Enemies?. Genes.

[52] De Filippis F., Pellegrini N., Vannini L., Jeffery I.B., La Storia A., Laghi L., Serrazanetti D.I., Di Cagno R., Ferrocino I., Lazzi C. (2015). High-level adherence to a Mediterranean diet beneficially impacts the gut microbiota and associated metabolome. Gut.

[53] Tomova A., Bukovsky I., Rembert E., Yonas W., Alwarith J., Barnard N.D., Kahleova H. (2019). The Effects of Vegetarian and Vegan Diets on Gut Microbiota. Front. Nutr..

[54] Vetrani C., Costabile G., Luongo D., Naviglio D., Rivellese A.A., Riccardi G., Giacco R., Information P.E.K.F.C. (2016). Effects of whole-grain cereal foods on plasma short chain fatty acid concentrations in individuals with the metabolic syndrome. Nutrients.

[55] Kovatcheva-Datchary P., Nilsson A.C., Akrami R., Lee Y.S., De Vadder F., Arora T., Hallén A., Martens E., Björck I., Bäckhed F. (2015). Dietary Fiber-Induced Improvement in Glucose Metabolism Is Associated with Increased Abundance of Prevotella. Cell Metab..

[56] Jefferson A., Adolphus K. (2019). The Effects of Intact Cereal Grain Fibers, Including Wheat Bran on the Gut Microbiota Composition of Healthy Adults: A Systematic Review. Front. Nutr..

[57] Si X., Shang W., Zhou Z., Shui G., Lam S.M., Blanchard C., Strappe P., Blanchard C. (2018). Gamma-aminobutyric Acid Enriched Rice Bran Diet Attenuates Insulin Resistance and Balances Energy Expenditure via Modification of Gut Microbiota and Short-Chain Fatty Acids. J. Agric. Food Chem..

[58] Rinninella E., Cintoni M., Raoul P., Lopetuso L.R., Scaldaferri F., Pulcini G., Miggiano G.A.D., Gasbarrini A., Mele M.C. (2019). Food Components and Dietary Habits: Keys for a Healthy Gut Microbiota Composition. Nutrients.

[59] Chassaing B., Koren O., Goodrich J.K., Poole A., Srinivasan S., Ley R.E., Gewirtz A.T. (2015). Dietary emulsifiers impact the mouse gut microbiota promoting colitis and metabolic syndrome. Nature.

[60] Laudisi F., Stolfi C., Monteleone G. (2019). Impact of Food Additives on Gut Homeostasis. Nutrients.

[61] Flint H.J., Bayer E., Rincon M.T., Lamed R., White B.A. (2008). Polysaccharide utilization by gut bacteria: Potential for new insights from genomic analysis. Nat. Rev. Genet..

[62] Lee W.-J., Hase K. (2014). Gut microbiota–generated metabolites in animal health and disease. Nat. Methods.

[63] Rooks M.G., Garrett W.S. (2016). Gut microbiota, metabolites and host immunity. Nat. Rev. Immunol..

[64] Lin L., Zhang J. (2017). Role of intestinal microbiota and metabolites on gut homeostasis and human diseases. BMC Immunol..

[65] Cai Y., Folkerts J., Folkerts G., Maurer M., Braber S. (2019). Microbiota-dependent and -independent effects of dietary fiber on human health. Br. J. Pharmacol..

[66] Rios-Covian D., Ruas-Madiedo P., Margolles A., Gueimonde M., Reyes-Gavilán C.G.D.L., Salazar N. (2016). Intestinal Short Chain Fatty Acids and their Link with Diet and Human Health. Front. Microbiol..

[67] Besten G.D., Van Eunen K., Groen A.K., Venema K., Reijngoud D.-J., Bakker B. (2013). The role of short-chain fatty acids in the interplay between diet, gut microbiota, and host energy metabolism. J. Lipid Res..

[68] Smith E.A., Macfarlane G.T. (1998). Enumeration of amino acid fermenting bacteria in the human large intestine: Effects of pH and starch on peptide metabolism and dissimilation of amino acids. FEMS Microbiol. Ecol..

[69] Sivaprakasam S., Bhutia Y.D., Yang S., Ganapathy V. (2017). Short-Chain Fatty Acid Transporters: Role in Colonic Homeostasis. Compr. Physiol..

[70] Høverstad T., Midtvedt T. (1986). Short-Chain Fatty Acids in Germfree Mice and Rats. J. Nutr..

[71] Levrat M.-A., Demigné C., Rémésy C. (1991). High Propionic Acid Fermentations and Mineral Accumulation in the Cecum of Rats Adapted to Different Levels of Inulin. J. Nutr..

[72] Tan J., McKenzie C., Potamitis M., Thorburn A.N., Mackay C., Macia L. (2014). The Role of Short-Chain Fatty Acids in Health and Disease. Adv. Immunol..

[73] Brown A.J., Goldsworthy S.M., Barnes A.A., Eilert M.M., Tcheang L., Daniels D., Muir A.I., Wigglesworth M.J., Kinghorn I., Fraser N.J. (2002). The Orphan G Protein-coupled Receptors GPR41 and GPR43 Are Activated by Propionate and Other Short Chain Carboxylic Acids. J. Biol. Chem..

[74] Vinolo M.A., Ferguson G.J., Kulkarni S., Damoulakis G., Anderson K., Bohlooly-Y M., Stephens L.R., Hawkins P., Curi R. (2011). SCFAs Induce Mouse Neutrophil Chemotaxis through the GPR43 Receptor. PLoS ONE.

[75] Layden B.T., Angueira A.R., Brodsky M., Durai V., Lowe W.L. (2013). Short chain fatty acids and their receptors: New metabolic targets. Transl. Res..

[76] Thion M.S., Low N., Silvin A., Chen J., Grisel P., Schulte-Schrepping J., Blecher R., Ulas T., Squarzoni P., Hoeffel G. (2018). Microbiome Influences Prenatal and Adult Microglia in a Sex-Specific Manner. Cell.

[77] Bilotta A.J., Cong Y. (2019). Gut microbiota metabolite regulation of host defenses at mucosal surfaces: Implication in precision medicine. Precis. Clin. Med..

[78] Macia L., Tan J., Vieira A., Leach K., Stanley D., Luong S., Maruya M., McKenzie C.I., Hijikata A., Wong C.H. (2015). Metabolite-sensing receptors GPR43 and GPR109A facilitate dietary fibre-induced gut homeostasis through regulation of the inflammasome. Nat. Commun..

[79] Pluznick J.L. (2013). A novel SCFA receptor, the microbiota, and blood pressure regulation. Gut Microbes.

[80] Paassen N.B., Vincent A., Puiman P.J., Van Der Sluis M., Bouma J., Boehm G., Van Goudoever J.B., Van Seuningen I., Renes I.B. (2009). The regulation of intestinal mucin MUC2 expression by short-chain fatty acids: Implications for epithelial protection. Biochem. J..

[81] Zhao Y., Chen F., Wu W., Sun M., Bilotta A.J., Yao S., Xiao Y., Huang X., Eaves-Pyles T.D., Golovko G. (2018). GPR43 mediates microbiota metabolite SCFA regulation of antimicrobial peptide expression in intestinal epithelial cells via activation of mTOR and STAT3. Mucosal Immunol..

[82] Ohata A., Usami M., Miyoshi M. (2005). Short-chain fatty acids alter tight junction permeability in intestinal monolayer cells via lipoxygenase activation. Nutrients.

[83] Segain J.P., De La Blétière D.R., Bourreille A., Leray V., Gervois N., Rosales C., Ferrier L., Bonnet C., Blottière H.M., Galmiche J.P. (2000). Butyrate inhibits inflammatory responses through NFkappaB inhibition: Implications for Crohn’s disease. Gut.

[84] Alex S., Lange K., Amolo T., Grinstead J.S., Haakonsson A.K., Szalowska E., Koppen A., Mudde K., Haenen D., Al-Lahham S. (2013). Short-Chain Fatty Acids Stimulate Angiopoietin-Like 4 Synthesis in Human Colon Adenocarcinoma Cells by Activating Peroxisome Proliferator-Activated Receptor γ. Mol. Cell. Biol..

[85] Schönfeld P., Wojtczak L. (2016). Short- and medium-chain fatty acids in energy metabolism: The cellular perspective. J. Lipid Res..

[86] Magnúsdóttir S., Ravcheev D., De Crécy-Lagard V., Thiele I. (2015). Systematic genome assessment of B-vitamin biosynthesis suggests co-operation among gut microbes. Front. Genet..

[87] Tofalo R., Cocchi S., Suzzi G. (2019). Polyamines and Gut Microbiota. Front. Nutr..

[88] Strandwitz P., Kim K.H., Terekhova D., Liu J.K., Sharma A., Levering J., McDonald D., Dietrich D., Ramadhar T., Lekbua A. (2018). GABA-modulating bacteria of the human gut microbiota. Nat. Microbiol..

[89] Allis C.D., Jenuwein T. (2016). The molecular hallmarks of epigenetic control. Nat. Rev. Genet..

[90] Rozek L.S., Dolinoy D.C., A Sartor M., Omenn G.S. (2014). Epigenetics: Relevance and implications for public health. Annu. Rev. Public Health.

[91] Takahashi K. (2013). Influence of bacteria on epigenetic gene control. Cell. Mol. Life Sci..

[92] Alenghat T., Artis D. (2014). Epigenomic regulation of host-microbiota interactions. Trends Immunol..

[93] Hullar M.A.J., Fu B.C. (2014). Diet, the gut microbiome, and epigenetics. Cancer J..

[94] Krautkramer K., Kreznar J.H., Romano K.A., Vivas E.I., Barrett-Wilt G.A., Rabaglia M.E., Keller M.P., Attie A.D., Rey F.E., Denu J.M. (2016). Diet-Microbiota Interactions Mediate Global Epigenetic Programming in Multiple Host Tissues. Mol. Cell.

[95] Qin Y., Wade P.A. (2018). Crosstalk between the microbiome and epigenome: Messages from bugs. J. Biochem..

[96] Gerhauser C. (2018). Impact of dietary gut microbial metabolites on the epigenome. Philos. Trans. R. Soc. B Biol. Sci..

[97] Miro-Blanch J., Yanes O. (2019). Epigenetic Regulation at the Interplay Between Gut Microbiota and Host Metabolism. Front. Genet..

[98] Kumar H., Lund R., Laiho A., Lundelin K., Ley R.E., Isolauri E., Salminen S. (2014). Gut Microbiota as an Epigenetic Regulator: Pilot Study Based on Whole-Genome Methylation Analysis. mBio.

[99] Ramos-Molina B., Sánchez-Alcoholado L., Cabrera-Mulero A., López-Domínguez R., Carmona-Saez P., Garcia-Fuentes E., Moreno-Indias I., Tinahones F.J. (2019). Gut Microbiota Composition Is Associated With the Global DNA Methylation Pattern in Obesity. Front. Genet..

[100] Cortese R., Lu L., Yu Y., Ruden D., Claud E.C. (2016). Epigenome-Microbiome crosstalk: A potential new paradigm influencing neonatal susceptibility to disease. Epigenetics.

[101] Greenblum S., Turnbaugh P.J., Borenstein E. (2011). Metagenomic systems biology of the human gut microbiome reveals topological shifts associated with obesity and inflammatory bowel disease. Proc. Natl. Acad. Sci. USA.

[102] Rossi M., Amaretti A., Raimondi S. (2011). Folate Production by Probiotic Bacteria. Nutrients.

[103] Kok D., Steegenga W.T., McKay J.A. (2018). Folate and epigenetics: Why we should not forget bacterial biosynthesis. Epigenomics.

[104] Schaible T.D., Harris R.A., Dowd S.E., Smith C.W., Kellermayer R. (2011). Maternal methyl-donor supplementation induces prolonged murine offspring colitis susceptibility in association with mucosal epigenetic and microbiomic changes. Hum. Mol. Genet..

[105] Lin C.-C.J., Wang M.C. (2017). Microbial metabolites regulate host lipid metabolism through NR5A–Hedgehog signalling. Nature.

[106] Remely M., Aumueller E., Merold C., Dworzak S., Hippe B., Zanner J., Pointner A., Brath H., Haslberger A.G. (2014). Effects of short chain fatty acid producing bacteria on epigenetic regulation of FFAR3 in type 2 diabetes and obesity. Gene.

[107] Mariño E., Richards J.L., McLeod K.H., Stanley D., Yap Y.A., Knight J., McKenzie C., Kranich J., Oliveira A.C., Rossello F. (2017). Gut microbial metabolites limit the frequency of autoimmune T cells and protect against type 1 diabetes. Nat. Immunol..

[108] Felizardo R.J.F., De Almeida D.C., Pereira R.L., Watanabe I.K.M., Doimo N.T.S., Ribeiro W.R., Cenedeze M.A., Hiyane M.I., Amano M.T., Braga T. (2019). Gut microbial metabolite butyrate protects against proteinuric kidney disease through epigenetic- and GPR109a-mediated mechanisms. FASEB J..

[109] Donohoe D.R., Holley D., Collins L.B., Montgomery S.A., Whitmore A.C., Hillhouse A., Curry K.P., Renner S.W., Greenwalt A., Ryan E.P. (2014). A gnotobiotic mouse model demonstrates that dietary fiber protects against colorectal tumorigenesis in a microbiota- and butyrate-dependent manner. Cancer Discov..

[110] Paluszczak J., Krajka-Kuźniak V., Baer-Dubowska W. (2010). The effect of dietary polyphenols on the epigenetic regulation of gene expression in MCF7 breast cancer cells. Toxicol. Lett..

[111] Remely M., Ferk F., Sterneder S., Setayesh T., Roth S., Kepcija T., Noorizadeh R., Rebhan I., Greunz M., Beckmann J. (2017). EGCG Prevents High Fat Diet-Induced Changes in Gut Microbiota, Decreases of DNA Strand Breaks, and Changes in Expression and DNA Methylation ofDnmt1andMLH1in C57BL/6J Male Mice. Oxid. Med. Cell. Longev..

[112] Pan P., Lam V., Salzman N., Huang Y.-W., Yu J., Zhang J., Wang L.-S. (2017). Black Raspberries and Their Anthocyanin and Fiber Fractions Alter the Composition and Diversity of Gut Microbiota in F-344 Rats. Nutr. Cancer.

[113] Meeran S.M., Patel S.N., Tollefsbol T.O. (2010). Sulforaphane Causes Epigenetic Repression of hTERT Expression in Human Breast Cancer Cell Lines. PLoS ONE.

[114] Takahashi K., Sugi Y., Nakano K., Tsuda M., Kurihara K., Hosono A., Kaminogawa S. (2011). Epigenetic Control of the Host Gene by Commensal Bacteria in Large Intestinal Epithelial Cells*. J. Biol. Chem..

[115] Poupeau A., Garde C., Sulek K., Citirikkaya K., Treebak J.T., Arumugam M., Simar D., Olofsson L., Bäckhed F., Barrès R. (2018). Genes controlling the activation of natural killer lymphocytes are epigenetically remodeled in intestinal cells from germ-free mice. FASEB J..

[116] Pan W.-H., Sommer F., Falk-Paulsen M., Ulas T., Best P., Fazio A., Kachroo P., Luzius A., Jentzsch M., Rehman A. (2018). Exposure to the gut microbiota drives distinct methylome and transcriptome changes in intestinal epithelial cells during postnatal development. Genome Med..

[117] Kellermayer R., Dowd S.E., Harris R.A., Balasa A., Schaible T.D., Wolcott R.D., Tatevian N., Szigeti R., Li Z., Versalovic J. (2011). Colonic mucosal DNA methylation, immune response, and microbiome patterns in Toll-like receptor 2-knockout mice. FASEB.

[118] Maekita T. (2006). High Levels of Aberrant DNA Methylation in Helicobacter pylori-Infected Gastric Mucosae and its Possible Association with Gastric Cancer Risk. Clin. Cancer Res..

[119] Krautkramer K., Dhillon R.S., Denu J.M., Carey H.V. (2017). Metabolic programming of the epigenome: Host and gut microbial metabolite interactions with host chromatin. Transl. Res..

[120] Smith P.M., Howitt M.R., Panikov N., Michaud M., Gallini C.A., Bohlooly-Y M., Glickman J.N., Garrett W.S. (2013). The Microbial Metabolites, Short-Chain Fatty Acids, Regulate Colonic Treg Cell Homeostasis. Science.

[121] Chatila T., Park J., Kim M., Kang S.G., Jannasch A.H., Cooper B., Patterson J., Kim C.H. (2015). Faculty of 1000 evaluation for Short-chain fatty acids induce both effector and regulatory T cells by suppression of histone deacetylases and regulation of the mTOR-S6K pathway. Mucosal Immunol..

[122] Zeng X., Sunkara L., Jiang W., Bible M., Carter S., Ma X., Qiao S., Zhang G. (2013). Induction of Porcine Host Defense Peptide Gene Expression by Short-Chain Fatty Acids and Their Analogs. PLoS ONE.

[123] Davie J.R. (2003). Inhibition of histone deacetylase activity by butyrate. J. Nutr..

[124] Vinolo M.A., Rodrigues H.G., Nachbar R.T., Curi R. (2011). Regulation of inflammation by short chain fatty acids. Nutrients.

[125] Nancey S., Bienvenu J., Coffin B., Andre F., Descos L., Flourie B. (2002). Butyrate strongly inhibits in vitro stimulated release of cytokines in blood. Dig. Dis. Sci..

[126] Berni Canani R., Di Costanzo M., Leone L. (2012). The epigenetic effects of butyrate: Potential therapeutic implications for clinical practice. Clin. Epigenetics.

[127] Yu D.C., Waby J., Chirakkal H., Staton C.A., Corfe B.M. (2010). Butyrate suppresses expression of neuropilin I in colorectal cell lines through inhibition of Sp1 transactivation. Mol. Cancer.

[128] Donohoe D.R., Collins L.B., Wali A., Bigler R., Sun W., Bultman S.J. (2012). The Warburg effect dictates the mechanism of butyrate-mediated histone acetylation and cell proliferation. Mol. Cell.

[129] Röth D., Chiang A.J., Hu W., Gugiu G.B., Morra C.N., Versalovic J., Kalkum M. (2019). Two-carbon folate cycle of commensal Lactobacillus reuteri 6475 gives rise to immunomodulatory ethionine, a source for histone ethylation. FASEB J..

[130] Kuang Z., Wang Y., Li Y., Ye C., Ruhn K.A., Behrendt C.L., Olson E.N., Hooper L.V. (2019). The intestinal microbiota programs diurnal rhythms in host metabolism through histone deacetylase 3. Science.

[131] Kubinak J.L., Stephens W.Z., Soto R., Petersen C., Chiaro T., Gogokhia L., Bell R., Ajami N.J., Petrosino J.F., Morrison L. (2015). MHC variation sculpts individualized microbial communities that control susceptibility to enteric infection. Nat. Commun..

[132] Clark A., Mach N. (2017). The Crosstalk between the Gut Microbiota and Mitochondria during Exercise. Front. Physiol..

[133] Borra M.T., Smith B., Denu J.M. (2005). Mechanism of Human SIRT1 Activation by Resveratrol. J. Biol. Chem..

[134] Chaplin A., Carpéné C., Mercader J. (2018). Resveratrol, Metabolic Syndrome, and Gut Microbiota. Nutrients.

[135] Schmeck B., Beermann W., Van Laak V., Zahlten J., Opitz B., Witzenrath M., Hocke A.C., Chakraborty T., Kracht M., Rosseau S. (2005). Intracellular bacteria differentially regulated endothelial cytokine release by MAPK-dependent histone modification. J. Immunol..

[136] Xia G., Schneider-Stock R., Diestel A., Habold C., Krueger S., Roessner A., Naumann M., Lendeckel U. (2008). Helicobacter pylori regulates p21WAF1 by histone H4 acetylation. Biochem. Biophys. Res. Commun..

[137] Ridlon J.M., Kang D.J., Hylemon P.B., Bajaj J.S. (2014). Bile acids and the gut microbiome. Curr. Opin. Gastroenterol..

[138] Ramos-Molina B., Queipo-Ortuño M.I., Lambertos A., Tinahones F.J., Peñafiel R. (2019). Dietary and Gut Microbiota Polyamines in Obesity- and Age-Related Diseases. Front. Nutr..

[139] Lee W.J., Shim J.-Y., Zhu B.T. (2005). Mechanisms for the Inhibition of DNA Methyltransferases by Tea Catechins and Bioflavonoids. Mol. Pharmacol..

[140] Roager H.M., Licht T.R. (2018). Microbial tryptophan catabolites in health and disease. Nat. Commun..

[141] Paul B., Barnes S., Demark-Wahnefried W., Morrow C.D., Salvador C., Skibola C.F., Tollefsbol T.O. (2015). Influences of diet and the gut microbiome on epigenetic modulation in cancer and other diseases. Clin. Epigenetics.

[142] Jones S.E., Paynich M.L., Kearns D.B., Knight K.L. (2014). Protection from intestinal inflammation by bacterial exopolysaccharides. J. Immunol..

[143] Mimee M., Citorik R.J., Lu T.K. (2016). Microbiome therapeutics—Advances and challenges. Adv. Drug Deliv. Rev..

[144] Cani P.D. (2018). Human gut microbiome: Hopes, threats and promises. Gut.

[145] Bhat M.I., Kapila R. (2017). Dietary metabolites derived from gut microbiota: Critical modulators of epigenetic changes in mammals. Nutr. Rev..

